# Does a pulmonary rehabilitation based ERAS program (PREP) affect pulmonary complication incidence, pulmonary function and quality of life after lung cancer surgery? Study protocol for a multicenter randomized controlled trial

**DOI:** 10.1186/s12890-020-1073-6

**Published:** 2020-02-18

**Authors:** Yu Zheng, Mao Mao, Meifang Ji, Qiugang Zheng, Liang Liu, Zhigang Zhao, Haiming Wang, Xiangyang Wei, Yulong Wang, Jiamin Chen, Huiqing Zhou, Qiaoqiao Liang, Ying Chen, Xintong Zhang, Lu Wang, Yihui Cheng, Xiu Zhang, Meiling Teng, Xiao Lu

**Affiliations:** 1 Department of Rehabilitation Medicine, the First Affiliated Hospital of Nanjing Medical University, No.300 Guangzhou Road, Nanjing, 210029 China; 2 Department of Rehabilitation Therapy, the Second Affiliated Hospital of Hainan Medical University, No. 368 Yehai Road, Haikou, 570100 China; 3 Department of Rehabilitation Medicine, Nanjing Drum Tower Hospital, the Affiliated Hospital of Nanjing University Medical School, No. 321 Zhongshan Road, Nanjing, 210008 China; 4 Department of Rehabilitation Medicine, the First Affiliated Hospital of Zhengzhou University, No. 1 East of Jianshe Road, Zhengzhou, 450052 China; 5 Department of Rehabilitation Medicine, Shenzhen Dapeng New District Nan’ao People’s Hospital, No. 6 Renmin Road, Dapeng New District, Shenzhen, 518000 China; 6 Department of Rehabilitation Therapy, Taizhou Enze Medical Center, Enze Hospital, No. 1 East of Tongyang Road, Taizhou, 318050 China

**Keywords:** Pulmonary rehabilitation, Lung cancer surgery, Enhanced recovery after surgery, Post-operative complication incidence, Pulmonary function, Health-related quality of life, Multicenter randomized controlled trial

## Abstract

**Background:**

Lung cancer surgery is associated with a high incidence of postoperative pulmonary complications (PPCs). Preliminary evidence suggests that ERAS processes can reduce overall incidence of PPCs as short- and long-term recovery improved by supporting units to adopt evidence-based care. However, the evidence is inconclusive due to insufficient high-level studies in this research field. No well-designed, adequately powered, randomized controlled trials (RCTs) have investigated the effects of pulmonary rehabilitation based ERAS program (PREP) on post-operative pulmonary complications, pulmonary function, and health related quality of life following lung cancer surgery.

**Methods:**

The PREP trial is a pragmatic, investigator-initiated, multi-center, randomized controlled, parallel group, clinical trial. Five hundred patients scheduled for minimally invasive pulmonary resection at six hospitals in China will be randomized with concealed allocation to receive either i) a pre-operative assessment and an information booklet or ii) a pre-operative assessment, an information booklet, plus an additional education, a 30-min pulmonary rehabilitation training session and the post-operative pulmonary rehabilitation program. The primary outcome is incidence of PPCs defined with the Melbourne Group Scale diagnostic scoring tool. Secondary outcomes include incidence of cardiopulmonary and other complications, pulmonary function, cardiopulmonary endurance, muscle strength, activity level, health-related quality of life (HRQoL), pre- and post-operative hospital length of stay (LOS), and total hospital LOS.

**Discussion:**

The PREP trial is designed to verify the hypothesis that pulmonary rehabilitation based ERAS program reduces incidence of PPCs and improves pulmonary function and HRQoL in patients following lung cancer surgery. This trial will furthermore contribute significantly to the limited knowledge about the pulmonary rehabilitation based ERAS program following lung cancer surgery, and may thereby form the basis of future recommendations in the surgical community.

**Trial registration:**

Chinese Clinical Trial Registry: ChiCTR1900024646, 21 July 2019.

## Background

Minimally invasive pulmonary resection is currently the most frequent and effective intervention for lung cancer. However, lung cancer surgery is associated with a high incidence of postoperative pulmonary complications (PPCs) with a reported incidence of 15.8–31.7% based on large sample studies [1, 2]. This is higher than the incidence for other major surgical procedures such as orthopedic surgery (0.3–3.6%), cardiac surgery (1.5–28.4%), upper and lower abdominal surgery (1.8–23.7% and 1.9–4.3%) [3–16]. The term PPC is defined as clinically significant respiratory complication or dysfunction requiring medical and rehabilitation intervention. Although the causes of PPCs are multifactorial, surgical procedure induced systemic inflammatory and catabolic response have been proposed as major contributing factors in the development of PPCs, leading to the changes in the short-term activity ability, and long-term health-related quality of life (HRQoL) and mortality after lung cancer surgery [17].

Enhanced Recovery After Surgery (ERAS) practices improve the opportunity for rapid, uncomplicated recovery after surgery with both short- and long-term benefits for patients [18]. Data suggest that complication rates can be reduced by 10–50% or more with supporting units to adopt evidence-based EARS care [19–22]. Further data also showed that not only were overall complications reduced, but the most severe complications, which resulted in decreased rates of reoperation or admission to the intensive care unit (ICU) as mortality improved [20, 23]. Recently, ERAS principles have been applied across most surgical specialties. After the ERAS Society was formed in 2010, the Society published a series of guidelines and recommendations on colonic resection, rectal resection and pancreaticoduodenectomy in 2012, cystectomy and gastric resection in 2015, bariatric surgery, liver resection, and head and neck cancer surgery in 2016, breast reconstruction in 2017 [18]. Nonetheless, the ERAS guideline for thoracic surgery is still under production, revealing that there is a paucity of quality resear on adopting these principles in this field. To date, no multicenter randomized controlled trials (RCTs) support the provision of pulmonary rehabilitation based ERAS program (PREP) on reducing PPCs and shortening recovery time, which translate to a substantial improvement of quality of life (QoL) following the ERAS principle. Considering the high incidence of PPCs following lung cancer surgery, ongoing innovation must move from guessing to knowing the outcomes though adequately powered research. Therefore, a well-designed, multicenter RCT is needed to determine the clinical effects of pulmonary rehabilitation based ERAS program on reducing incidence of PPCs following lung cancer surgery. The results of the current trial may fill the blank of applying ERAS program in this field and may contribute to the construction of ERAS guideline on lung cancer surgery.

### Trial objectives

The primary objective of the PREP trial is to investigate the effects of PREP, specifically pre-operative education combined with post-operative pulmonary rehabilitation, on the incidence of PPCs following lung cancer surgery. Secondary objectives are to estimate the effects of PREP on cardiopulmonary complications, other complications pulmonary function, cardiopulmonary endurance, muscle strength, activity level and HRQoL, pre-operative and post-operative hospital LOS, and total hospital LOS.

## Methods/design

### Trial design

The PREP trial is a pragmatic, investigator-initiated, multi-center, randomized controlled, parallel group, clinical trial. Table 1 shows the overview of the trial methods and design.

**Table 1:** World Health Organization (WHO) Trial Registration Data Set for PREP trial

Data category	Information
Primary registry and trial identifying number	Chinese Clinical Trial Registry number: ChiCTR1900024646
Date of registration in primary registry	21/07/2019
Secondary identifying numbers	N/A
Trial protocol version	Version 1
Source(s) of monetary or material support	Clinical research grant from the Department of Rehabilitation Medicine in the First Affiliated Hospital of Nanjing Medical University (1,200,000 CNY)
Primary sponsor	N/A
Secondary sponsor	N/A
Contact for public queries	XL, luxiao1972@163.com
Contact for scientific queries	XL, luxiao1972@163.com
Public title	Pre- and post-operative physiotherapy for the prevention of complications and the improvement of pulmonary function and quality of life after lung cancer surgery: a multi-center randomized controlled trial
Scientific title	Pre- and post-operative physiotherapy for the prevention of complications and the improvement of pulmonary function and quality of life after lung cancer surgery: a multi-center randomized controlled trial
Countries of recruitment	China
Health condition(s) or problem(s) studied	Complication incidence, pulmonary function and quality of life following lung cancer surgery
Intervention(s)	Active comparator: Education booklet combined with a 30-min pre-operative pulmonary rehabilitation training session and daily post-operative pulmonary rehabilitation
	Placebo comparator: Education booklet
Key inclusion and exclusion criteria	Ages eligible for study: 18-80 yr
	Sexes eligible for study: both
	Accepts health volunteers: No
	Inclusion criteria: (i) diagnose with lung cancer through medical history, clinical symptoms, radiographic assessment and pathological results; (ii) Karnfsky ≥60 and estimated survival period > 6 months; (iii) to undergo minimally invasive lung cancer surgery (namely the video-assisted thoracoscopic surgery, VATS); (iv) with normal cognitive function and being able to cooperate with the rehab training; (v) the participation in the PREP trial and sign the informed consent form
	Exclusion criteria: (i) refuse the randomization; (ii) with severe limb dysfunction or systemic diseases and being unable to cooperate with the rehab training; (iii) with severe cognitive and mental dysfunctions; (iv) participated in other trials previously
Study type	Type: Pragmatic, investigator-initiated, multi-center, randomized controlled, parallel group, clinical trial
	Allocation: Concealed randomization
	Intervention model: Parallel assignment
	Masking: Assessor, surgeon, data analyst, and statistician blinded
	Primary purpose: Prevention and improvement
	Phase: Phase III
Date of first enrolment	1/8/2019
Target sample size	500
Recruitment status	Recruiting
Primary outcome(s)	Post-operative pulmonary complications incidence
Key secondary outcomes	Pulmonary function, six-minute walking test, health-related quality of life

### Trial setting

The six participating centers from different regions generally represent the whole picture of China. The First Affiliated Hospital of Nanjing Medical University (Nanjing in Eastern China) is a 3700-bed metropolitan, primary referral hospital; the Second Affiliated Hospital of Hainan Medical University (Haikou in Southern China) is a 2100-bed coastal, primary referral hospital; Nanjing Drum Tower Hospital, the Affiliated Hospital of Nanjing University Medical School, (Nanjing in Eastern China) is a 3000-bed metropolitan, primary referral hospital; the First Affiliated Hospital of Zhengzhou University (Zhengzhou in central China) is a 7000-bed inner-regional, primary referral hospital; Shenzhen Dapeng New District Nan’ao People’s Hospital (Shenzhen in Southern China) is a 300-bed coastal, secondary referral hospital and Taizhou Enze Medical Center, Enze Hospital (Taizhou in Eastern China) is a 1000-bed medium-sized, primary referral hospital. The First Affiliated Hospital of Nanjing Medical University and the Second Affiliated Hospital of Hainan Medical University have implemented Enhanced Recovery After Surgery (ERAS) guidelines to all surgical units. Relevant medical staff from the six participating centers have been trained with the standardized procedure of enrollment, randomization, rehabilitation intervention, assessment and data collection at the kick-off meeting.

### Eligibility criteria

The target population for this study are those who met the consolidated inclusion criteria: (i) diagnose with lung cancer through medical history, clinical symptoms, radiographic assessment and pathological results; (ii) aged 18–80; (iii) Karnfsky ≥60 and estimated survival period > 6 months; (iv) to undergo minimally invasive lung cancer surgery (namely the video-assisted thoracoscopic surgery, VATS); (v) with normal cognitive function and being able to cooperate with the rehab training; (vi) the participation in the PREP trial and sign the consent form [24–27].

The exclusion criteria are as follows: (i) refuse the randomization; (ii) with severe limb dysfunction or systemic diseases and being unable to cooperate with the rehab training; (iii) with severe cognitive and mental dysfunctions; (iv) participated in other trials previously [28–30].

The institutional review board in the First Affiliated Hospital of Nanjing Medical University has approved the trial with the protocol reference of 2019-SR-360. The trial will be conducted in accordance with the Declaration of Helsinki and was registered on 21 July 2019 at the Chinese Clinical Trial Registry (http://www.chictr.org.cn): ChiCTR1900024646.

### Recruitment, randomization and allocation

Eligible patients potentially undergoing lung cancer surgery at the participating centers will be assessed by a multi-disciplinary team consisting of a registered nurse, anesthetist, and surgeon from the admitting surgical team one to 2 weeks prior to their operation. Face-to-face pre-operative education in terms of the surgical process, pain management, postoperative drips and drains, and expected recovery process will be delivered.

Six sets of allocation sequence is determined by a web-based computer generated (http://www.randomizer.org/) random number table (Odd = intervention, Even = control). These tables are then sealed in six sets of 150 sequentially numbered (1 to 150) opaque envelopes, each containing an allocation card, prepared by a medical staff masked to the trial [31].

Eligible patients will be provided with a sheet including trial information about the randomization process, the discrepant rehabilitation plans, the surgical process, pain management, postoperative drips and drains, and expected recovery process. Those agreeing will sign a consent form as required by the ethics committee and in accordance with the Declaration of Helsinki.

**Fig. 1:**
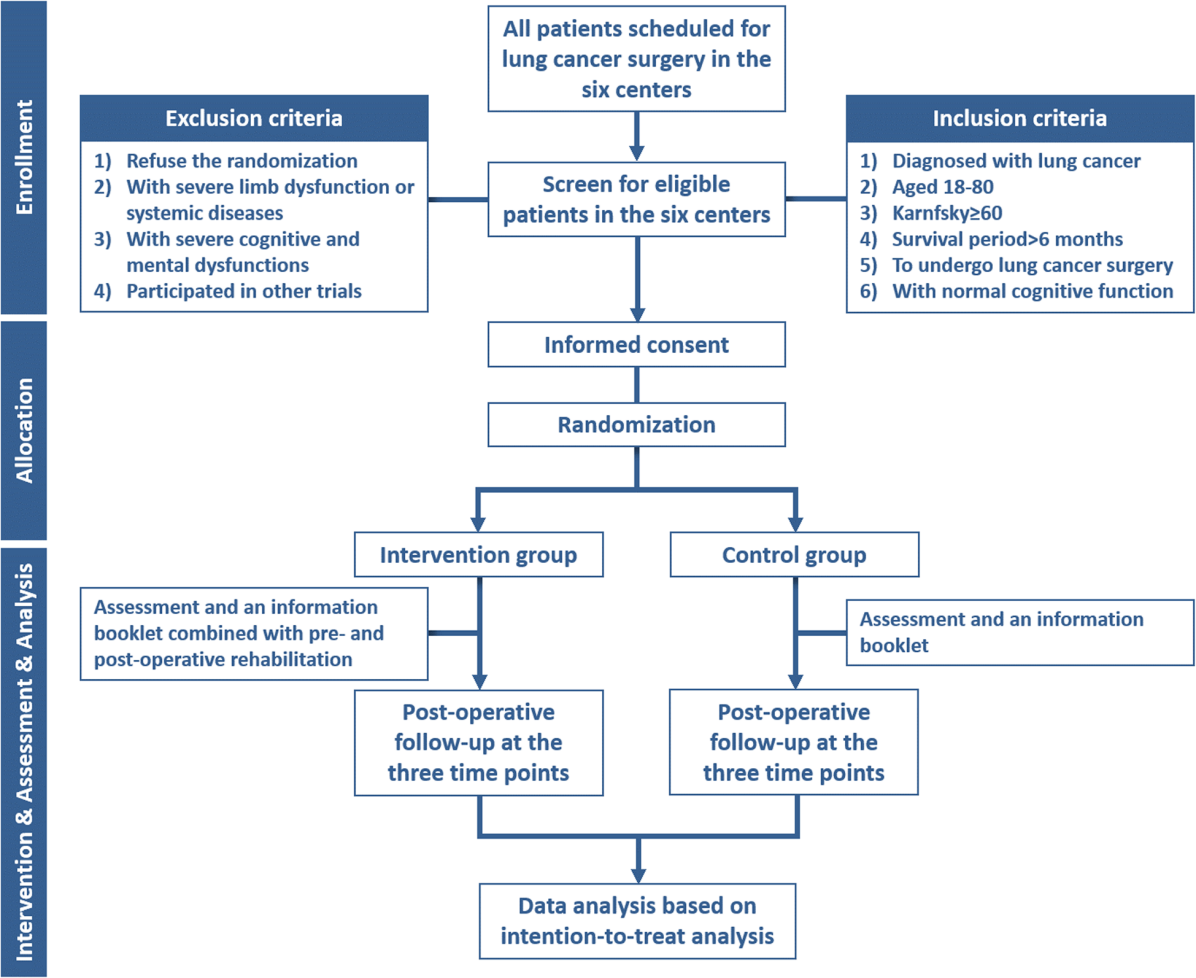
Flow chart of the PREP trial

Once informed consent form has been signed, patients will be allocated to one of the two experimental groups, by opening the sequentially numbered opaque envelopes, to receive 1) a pre-operative assessment and an information booklet (control) or 2) a pre-operative assessment, an information booklet, plus an additional education, a 30-min pulmonary rehabilitation training session and the post-operative pulmonary rehabilitation program (intervention). A trial participation sticker containing patient details will be attached on the opened envelope which will be filed securely with the informed consent form. Figure 1 demonstrates the overview of the recruitment, randomization and allocation based on the CONSORT principle.

### Blinding

Trial assistants and clinic nurses at each study center will be masked to the group allocation pre- and post-operatively. Due to the nature of the intervention, the patients cannot be blinded to the group allocation. In each center, two groups of physiotherapists will be assigned to the intervention group or the control group to maximally avoid performance bias. The trial administrators at each study center who are aware of the group allocation will assign patients in the intervention group to one medical unit whereas the control group to the other unit. The medical staff including assessors, surgeons, data analysts, and statisticians will be blinded to the group allocation.

### Interventions

#### Control group

At their admission, patients in the control group will be provided with a color booklet containing information about the general procedure of lung cancer surgery, strategies of pain management, post-operative recovery process and potential complications, and the necessity and the importance of early ambulation and self-directed pulmonary rehabilitation to the post-operative recovery. Detailed written and pictorial instructions will direct the patients to correctly perform deep breathing and coughing. In this case, no verbal education or guided training will be provided by a physiotherapist.

#### Exercise group

Apart from the booklet, patients in the exercise group will be provided with an additional 30-min training session guided by a physiotherapist. This session contains the verbal education and guided training of 1) diaphragmatic breathing, diaphragm muscles could be strengthened through this exercise (15 repetitions each set following 2-3 min rest for three sets). The patients inhale slowly to the maximum lung capacity through nose, and hold the breath for 2–3 s, then exhale slowly through tips with the abdominal muscles tighten; 2) effective coughing, the patient is requested to perform a slow-flow breath to maximum inspiratory capacity followed by a forced cough with the glottis opening. One hand of the patient or the physiotherapist presses over on the surgical region to support the wound and to encourage greater expiratory force; and 3) aerobic ambulation, the patient is encouraged to perform indoor walking and it is aiming for an initial intensity of 50–70% VO_2_ peak based on the results of 6MWT tested at the baseline with an increase of 10–15% VO_2_ peak per day according to the condition of each individual. They are requested to slow down or stop walking with the occurrence of chest tightness or breath shortness. Outside these assisted sessions, participants will be advised to walk or exercise by their bedside as frequently as they are able. Patients are requested to perform self-directed training till the operation day. After the surgery, a physiotherapist will assist the patients to walk as soon as possible on the first post-operative day. The diaphragmatic breathing, effective coughing and aerobic ambulation will be performed till the discharge day under the supervision of the physiotherapist to maximally avoid adverse events [32–34].

### Withdrawal from trial

Patients will be withdrawn for either of the following: (i) the patient makes such a request; (ii) the patient develops a serious disease, such as heart disease or stroke, and continuing their participation becomes inappropriate in the opinion of the investigators; (iii) an severe adverse event related to the pulmonary rehabilitation program occurs or (iv) with poor compliance to the training protocol.

### Outcome measures

#### Primary outcome

The primary outcome of the PREP trial is the incidence of PPCs within the first 14 post-operative days. PPCs is defined with the Melbourne Group Scale (MGS) diagnostic scoring tool, which is reliable and valid following thoracic surgery with high inter-rater reliability [35–37]. A PPC will be diagnosed when the following four or more factors are identified: 1) new abnormal breath sounds different to pre-operative; 2) chest X-ray findings of atelectasis or consolidation; 3) raised white cell count (WCC) (> 11 × 10^9^/L); 4) temperature > 38 °C; 5) purulent sputum differing from preoperative status; 6) signs of infection on sputum culture; 7) pulse oximetry oxygen saturation (SpO_2_) < 90% without oxygen therapy; and 8) pneumonia diagnosed based on physician’s experience [38]. When a positive diagnosis of a PPC is confirmed, the patient will then receive specific respiratory interventions provided by the doctors from Department of Respiration.

Patients will be assessed for PPCs at each prospective day by a blinded assessor. Medical documents including radiologist reports and laboratory sheets will be reviewed as references. A log-sheet will be provided to the patients on their discharge day to record any self-identified respiratory symptoms in the following consecutive days till Day 14. These data may sever as references for diagnosis of PPCs during their stay at home. The patients will be requested to submit the log-sheets to the assessor for recording on their follow up days. They will be suggested to readmitted to the hospital in case of any severe symptom occurs.

#### Secondary outcomes

Secondary outcomes are listed as follows:

1. Cardiopulmonary complications, other than those defined in the MGS, include atelectasis, respiratory failure, pleural effusion, pneumothorax, pulmonary embolism, arrhythmia, heart failure and thoracic hemorrhage;
2. Other complications include hypovolemia, wound lacerations, wound hemorrhage, wound dehiscence, wound infection, urinary tract infection, sepsis, delirium, reintubation and fall;
3. Pulmonary function will be reflected by forced vital capacity (FVC), forced expiratory volume in the first second (FEV_1_), ratio between FEV_1_ and FVC (FEV_1_/FVC), peak expiratory flow (PEF) [39, 40];
4. Cardiopulmonary endurance will be reflected by the 6-min walking test (6MWT) [27];
5. Muscle strength will be reflected by maximum grip strength as measured on the dominant hand using a calibrated hand dynamometer [41];
6. Activity level will be reflected by self-reported metabolic equivalents (METs) [42];
7. Health-related quality of life (HRQoL) will be evaluated with SF-12 [43];
8. Time in day for the pre- and post- operative application of antibiotics;
9. Pre- and post-operative hospital LOS and total hospital LOS.

### Data collection

Data in terms of PPC defined in the MGS, cardiopulmonary complications (i.e. coronary heart disease, hypertension, COPD), other complications (i.e. diabetes), pulmonary function, cardiopulmonary endurance, muscle strength, activity level and HRQoL will be selectively collected on the admission day (baseline), on the discharge day (second follow-up), 2 weeks post operation (third follow-up) and 12 weeks post operation (forth follow-up). Scheduled data collection at each time point is presented in Table 2.

**Table 2:**
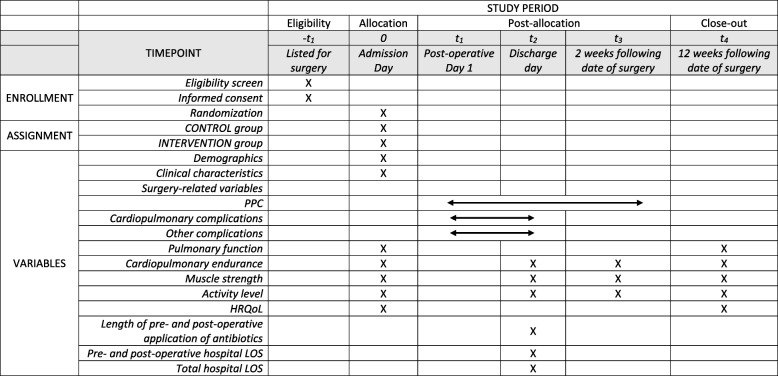
Scheduled events and timeline of PREP trial

*PPCs* post-operative pulmonary complications, *HRQoL* health-related quality of life, *LOS* length of stay

Apart from the above variables, demographics and clinical characteristics, collected directly from the patients or the medical documents, are as follows: center of recruitment, phone number, age, gender, height in centimeter, weight in kilogram, smoking history (never smoked, former smoker or current smoker), smoking index in average pack years, pre-operative comorbidities (cardiac disease, diabetes mellitus, hypertension, respiratory infection or others), pre- and post-operative hospital LOS and total hospital LOS.

Surgery-related variables will be collected from the anesthetic record, operation report and medical document: type of lung cancer according to the intra- or post-operative pathological examination, tumor location (left lower lobe, left upper lobe, right lower lobe, right middle lobe, right upper lobe) [44], surgical type (lobectomy, segmentectomy, pneumonectomy or sleeve) [36], length in minute of surgery, immediate post-operative location (surgical ward or ICU), post-operative antibiotic delivery (time in day and type of antibiotic delivered and reason to upgrade or downgrade), post-operative pain management (oral, intramuscular injection, intravenous administration or epidural administration), and postoperative daily drainage in milliliter.

### Sample size calculation

It has been reported that the absolute risk reduction in PPCs was of 20% by previous trials of preoperative education [45], and a post-operative PPC incidence of 32% was identified in patients with undergoing minimally invasive lung cancer surgery [2]. For the purposes of this trial, conservative goals (minimum 10% absolute risk reduction from a 20% baseline PPC risk) were set. A sample of 305 patients would have 80% power to detect a significant difference between groups (*p* = 0.05, two sided) with an 15% inflation to account for dropouts, non-compliance, and uncertainty of baseline risk, providing a final sample size of 500.

### Data management

Standardized case report forms (CRF) have been developed specifically for the trial. Completed forms are periodically sent by the participating centers to the coordinating center (the First Affiliated Hospital of Nanjing Medical University) for verification and data entry though a secured website. Local policy and national data protection guidance will be followed with study data anonymously recorded on a bespoke trial database using unique study identification numbers.

### Statistical methods

Backward stepwise regression will be applied to select adjustment covariates from covariates which may have impact on the prognostic strength and effect sizes between groups. These covariates include: age, gender, BMI, smoking history, smoking index, pre-operative comorbidities and physical status, pre-operative hospital LOS, application of prophylactic antibiotics, surgical type, tumor location, length in time of operation, ICU admission immediately following the surgery, mode of post-operative analgesia, type of lung cancer according to the intra- or post-operative pathological examination, time in day and type of post-operative antibiotic delivery, post-operative analgesia management, and postoperative daily drainage [46, 47].

For all outcomes we estimated differences in effect size between groups on an intention-to-treat basis. Multivariate robust random or mixed effects Poisson regression will be applied to estimate primary outcome efficacy and binomial secondary outcomes (cardiopulmonary complications and other complications). The time effect of day the PPC diagnosed between groups will be estimated with Cox proportional hazards regression with or without adjustment for covariates. Secondary outcomes with normal and abnormal distributions, including cardiopulmonary endurance, muscle strength, activity level, HRQoL, time in day of the application of antibiotics, pre- and post-operative hospital LOS and total hospital LOS, will be estimated for group differences with mixed effects ordered logistic regression and mixed effects linear regression.

Exploratory post hoc sensitivity adjusted analyses will be performed to determine the effect of specific covariates (discrepancy in experience grade of treating physiotherapist, hospital level, surgical category, tumor location, preoperative respiratory complication risk score, age and gender) across all primary and major secondary outcomes [48].

All analyses will be performed using Stata version 16 or later (StataCorp, College Station, TX, USA).

### Data monitoring

A data safety monitoring committee (DSMC) will be established to protect the validity and credibility of the trial according to the DAMOCLES Study Group’s recommendations [49]. The DSMC will meet periodically during the trial with the role to: monitor and review patient safety in the trial; request the conduct of interim data analyses (if required); and to review patient recruitment, accrual and withdrawal. The responsibility of the DSMC is not limited to provide recommendations about continuing or modifying the trial. The trial may be stopped by the DSMC if any severe adverse events are considered to have been caused by the pulmonary rehabilitation program or the assessments.

## Discussion

Following the changes in modern surgical care, interest in ERAS programs, which typically focus on minimizing pre-operative stress and improve the response to the post-operative stress, have grown substantially. The potential to significantly reduce the incidence of PPCs with easily provided interventions of pre- and post-operative rehabilitation therapy is appealing. Evidence suggests that change in clinical practice occurs 15 years after clear evidence is available [50]. There is an urgent need to support the medical and surgical community to implement new and better care. Following the guideline and recommendations in several surgical categories, unfortunately conclusive evidence of PREP on PPCs and other clinical outcomes is lacking.

The PREP trial is the first multicenter RCT designed to investigate whether pulmonary rehabilitation based ERAS program reduces the incidence of PPCs, and improves pulmonary function and HRQoL following lung cancer surgery. It has been widely recognized that a well-designed and powered multicenter RCT can provide the highest level of evidence to prove the effectiveness of certain interventions. However, performing a multicenter RCT may be with great difficulties. For example, the standardized intervention needs to be delivered across all the participating centers and the trial period. This can be addressed to train the physiotherapists in advance. The standard operation procedure (SOP) booklet and standard operation video have been drafted and recorded respectively. The physiotherapists from all the participating centers are required to adhere to the instruction as included within the SOP booklet and the digital video recording. Considering the discrepancy of learning curve and knowledge background across senior and junior physiotherapists, experience varies between participating centers, and will vary over time. We have not attempted to standardize these due to the feasibility of doing so across the six participating centers. Instead, the impact of potential confounders can be pooled and adjusted during statistical analysis. In addition, patient-oriented confounders, including timing of post-operative ambulation, pre-operative hospital LOS or post-operative analgesia management, have not been strictly controlled since this would be extremely difficult to achieve. Nonetheless, we believe that with effective randomization and blinding, it is reasonable to expect that patients in both groups will have an equal chance of exposure to these factors. Moreover, the differences in effect sizes of PREP versus control on all outcomes will be estimated with the intention-to-treat based different regression analysis which may be a complementary way to study new interventions rather than relying on simple Independent sample t-test or One-way ANOVA [18]. In similar with the placebo in the pharmaceutical research, the control group will be provided with educational booklet instead of no-intervention comparator. This action would be helpful to control for the Hawthorne effect and smooth the conduct of the PREP trial.

Objective and subjective parameters are both considered to include in the primary and secondary outcomes since in modern health care delivery it is essential to consider the impact of an intervention not only on objective incidence of PPCs or pulmonary function but on subjective patient-oriented HRQoL. As demonstrated previously, reduction in PPCs may influence HRQoL following discharge from hospital, particularly physical functioning domains [32, 51].. Therefore, four follow up time points will be set in the PREP trial to better understand the potential effects that the objective PPCs may have on the subjective HRQoL across the trial period.

To sum up, the PREP trial is a pragmatic, investigator-initiated, multi-center, randomized controlled, parallel group, clinical trial, designed to explore the hypothesis that pulmonary rehabilitation based ERAS program reduces incidence of post-operative pulmonary complications and improves pulmonary function and HRQoL in patients following lung cancer surgery.

## Trial status

The trial is ongoing and is actively enrolling. Enrollment of patients was initiated by the six participating centers on 1 August 2019. With an expected inclusion rate of 15 to 20 patients per month in total, an estimated recruitment period of 24–36 months is required.

## Data Availability

The datasets used and/or analyzed during the current study are available from the corresponding author on reasonable request.

## Abbreviations

6MWT: 6-min walking test
CRF: Case report forms
DSMC: Data safety monitoring committee
ERAS: Enhanced recovery after surgery
FEV_1_: Forced expiratory volume in the first second
FEV_1_/FVC: Ratio between FEV_1_ and FVC
FVC: Forced vital capacity
HRQoL: Health-related quality of life
ICU: Intensive care unit
LOS: Length of stay
METs: Metabolic equivalents
MGS: Melbourne Group Scale
PEF: Peak expiratory flow
PPCs: Postoperative pulmonary complications
PREP: Pulmonary rehabilitation based ERAS program
RCTs: Randomized controlled trials
SOP: Standard operation procedure
WCC: White cell count
WHO: World Health Organization
